# Verbal autopsy-based cause-specific mortality trends in rural KwaZulu-Natal, South Africa, 2000-2009

**DOI:** 10.1186/1478-7954-9-47

**Published:** 2011-08-05

**Authors:** Abraham J Herbst, Tshepiso Mafojane, Marie-Louise Newell

**Affiliations:** 1 Africa Centre for Health and Population Studies, University of KwaZulu-Natal, Somkhele, South Africa; 2 MRC Centre of Epidemiology for Child Health, UCL ICH, London, UK

## Abstract

**Background:**

The advent of the HIV pandemic and the more recent prevention and therapeutic interventions have resulted in extensive and rapid changes in cause-specific mortality rates in sub-Saharan Africa, and there is demand for timely and accurate cause-specific mortality data to steer public health responses and to evaluate the outcome of interventions. The objective of this study is to describe cause-specific mortality trends based on verbal autopsies conducted on all deaths in a rural population in KwaZulu-Natal, South Africa, over a 10-year period (2000-2009).

**Methods:**

The study used population-based mortality data collected by a demographic surveillance system on all resident and nonresident members of 12,000 households. Cause of death was determined by verbal autopsy based on the standard INDEPTH/WHO verbal autopsy questionnaire. Cause of death was assigned by physician review and the Bayesian-based InterVA program.

**Results:**

There were 11,281 deaths over 784,274 person-years of observation of 125,658 individuals between Jan. 1, 2000 and Dec. 31, 2009. The cause-specific mortality fractions (CSMF) for the population as a whole were: HIV-related (including tuberculosis), 50%; other communicable diseases, 6%; noncommunicable lifestyle-related conditions, 15%; other noncommunicable diseases, 2%; maternal, perinatal, nutritional, and congenital causes, 1%; injury, 8%; indeterminate causes, 18%. Over the course of the 10 years of observation, the CSMF of HIV-related causes declined from a high of 56% in 2002 to a low of 39% in 2009 with the largest decline starting in 2004 following the introduction of an antiretroviral treatment program into the population. The all-cause age-standardized mortality rate (SMR) declined over the same period from a high of 174 (95% confidence interval [CI]: 165, 183) deaths per 10,000 person-years observed (PYO) in 2003 to a low of 116 (95% CI: 109, 123) in 2009. The decline in the SMR is predominantly due to a decline in the HIV-related SMR, which declined in the same period from 96 (95% CI: 89, 102) to 45 (95% CI: 40, 49) deaths per 10,000 PYO.

There was substantial agreement (79% kappa = 0.68 (95% CI: 0.67, 0.69)) between physician coding and InterVA coding at the burden of disease group level.

**Conclusions:**

Verbal autopsy based methods enabled the timely measurement of changing trends in cause-specific mortality to provide policymakers with the much-needed information to allocate resources to appropriate health interventions.

## Background

The advent of the HIV pandemic and the more recent prevention and therapeutic interventions have resulted in extensive and rapid changes in cause-specific mortality rates in sub-Saharan Africa during the last two decades [1-3]. South Africa, in particular, with a severe HIV epidemic, experienced a steep rise in adult mortality during the 1990s and the early part of this decade [4,5] and is one of few countries where child mortality increased from the 1990 baseline [6]. In the past few years, evidence is emerging from South Africa [7,8] and elsewhere in sub-Saharan Africa [9-12] that HIV-related mortality is declining following the introduction of prevention and treatment programs. In South Africa, these changes have occurred against the backdrop of a steadily increasing noncommunicable disease burden [13] and high trauma-related mortality [14]. With limited reliable data as to whether HIV-related health care is flourishing to the detriment of non-HIV care, there is demand for timely and accurate cause-specific mortality data to steer public health responses and to evaluate the outcome of interventions [15].

Due to the lack of death registration systems in the majority of the world's poorest settings, verbal autopsy-based mortality surveillance has become one of the methods of choice to obtain the much needed cause-specific mortality data [15]. In particular, health and demographic surveillance sites have a role to play in this context to provide timely and accurate data [16]. Although South Africa has a functioning death registration system, the quality of cause of death data has been questioned [17,18] and data from demographic surveillance studies using verbal autopsies have contributed to determining cause-specific mortality rates [19-21] in the country.

Verbal autopsy (VA) methods have traditionally depended on physician assessment of the verbal autopsy interview data to determine a cause of death [22], but the efficiency, reliability, and repeatability of this approach have recently been questioned [22,23]. Alternative methods to determine the cause of death on the basis of a verbal autopsy interview have been developed [22,24], such as the InterVA (http://www.interva.net) system, which applies Bayes' theorem to derive probable causes of death from VA data [25].

Using data from a well-established longitudinal demographic surveillance, this study describes cause-specific mortality trends based on verbal autopsies conducted on all deaths in a rural population in KwaZulu-Natal, South Africa, over a 10-year period (2000-2009).

## Methods

### Study area and population

The Africa Centre for Health and Population Studies hosts a demographic surveillance program in the district of Umkhanyakude in the province of KwaZulu-Natal, South Africa [26]. Although it is largely rural, the demographic surveillance area (DSA), consisting of 435 square kilometers, also includes a township and periurban informal settlements. The population is characterized by high HIV prevalence [27] and incidence [28], but following the introduction of prevention of mother-to-child transmission of HIV infection (in 2001) and antiretroviral treatment and care (in 2004) programs, child [8] and adult [7] all-cause mortality have started to decline. The study population has comparatively high levels of cardiovascular risk factors [29] and high trauma-related mortality [30].

### Mortality data

The approximately 12,000 households in the DSA were visited thrice annually by fieldwork teams and all deaths were notified. Upon death notification, a trained nurse conducted an interview with the closest caregiver (parent or grandparent, 26%; spouse, child, or grandchild, 25%; other relative, 20%; sibling, 14%) of the deceased on average six months after the death. The nurses recorded a narrative of the circumstances leading up to the death and completed a questionnaire based on the standard INDEPTH/WHO verbal autopsy questionnaire [31,32]. Interviews were conducted in the local language and transcribed by the interviewer, after verbal consent. There were 228 (2%) refusals and 102 (0.9%) cases where a suitable interviewee could not be identified. A total of 13 nurses acted as interviewers over the course of the study; 80% of the interviews were conducted by six of these nurses. The majority of the nurses were graduate professional nurses; the remainder had two-year nursing diplomas. All were previously employed with local health services and received training in administering the verbal autopsy questionnaire.

Two methods were used to determine cause of death for each case: physician coding and an automated method using the InterVA v3 probabilistic verbal autopsy interpretation model. In the physician-coded method, two clinicians independently assigned cause of death on the basis of the information collected during the verbal autopsy and their clinical judgement. If consensus could not be reached between the physicians, the VA interview was refused, or no suitable interviewee could be found, the cause of death was recorded as "undetermined." A third clinician reviewed all cases and codified the causes of death using the International Classification of Diseases, 10^th ^revision (ICD-10)[33]. The ICD-10 codes were mapped into global burden of disease groups (Table 1) using the crosswalk in Lopez [34]. A total of 27 physicians were involved over the course of the study period (2000 to 2009) in assigning diagnoses, however, eight physicians were responsible for 79% of the recorded diagnoses. Complete cause of death coding for the physician-coded diagnoses was available for deaths between 2000 and the end of 2008.

**Table 1:** Burden of disease groups

Cause category	Abbreviation	Burden of disease codes
HIV-related causes	HIV-related	U003, U009

Other communicable diseases	Other CD	U005, U007, U008, U010, U015, U016, U017, U018, U020, U033, U037, U039

Maternal, perinatal, nutritional, and congenital causes	MPNC	U042, U043, U044, U045, U047, U048, U050, U051, U052, U054, U131, U136, U139, U140, U142

Noncommunicable lifestyle-related conditions	Lifestyle	U079, U105, U106, U107, U108, U109, U110, U112, U113, U114, U116, U117, U119, U120, U121, U122, U123

Other noncommunicable conditions	Other NCD	U059, U060, U061, U062, U063, U064, U065, U067, U068, U069, U070, U071, U072, U073, U074, U075, U076, U077, U078, U080, U081, U082, U084, U085, U086, U087, U096, U097, U124, U125, U130

Injuries	Injuries	U148, U150, U151, U152, U153, U154, U155, U157, U158

Indeterminate causes	Indeterminate	U000, Z900, Z993, Z994, Z997, Z998, Z999

The InterVA model is based on Bayesian calculations of probabilities that a particular death was due to particular causes, given a set of symptoms and circumstances associated with the death [25]. The verbal autopsy questionnaire data were converted into the 106 input indicators required by the InterVA probabilistic model using an SQL script. Cause of death categories were obtained by running InterVA in batch mode on the input indicators with malaria prevalence set to "low" and HIV prevalence set to "high." The 35 possible InterVA cause categories were mapped to the corresponding burden-of-disease codes to have cause categories comparable to the physician-coded diagnoses. InterVA produced up to three possible causes of death per case with a likelihood value between 0 and 1 for each cause. In cases where the likelihood values did not sum to 1 for a particular case, the difference between the sum of the likelihood values for the identified causes and 1 were allocated to the "indeterminate" cause. As recommended by the developers of InterVA, all identified causes were considered proportionate to their likelihood values in the rate calculations.

### Data analysis

Deaths and person-years of observation were aggregated annually for the period from Jan. 1, 2000, to Dec. 31, 2009, for all individuals in the study population.

Individuals contributed to the person-years denominator from Jan. 1, 2000, or from any later date of birth or in-migration, until Dec. 31, 2009, and they ceased to contribute to the denominator at death, termination of household membership, household out-migration, or the last surveillance visit in which household membership was confirmed. Thus, individuals who were previous homestead residents continued to be followed when they became nonresidents for as long as they remained a member of (retained links with) the household under surveillance. Out-migrants continued to be followed as nonresident household members in 83% of emigrations over the duration of the study.

We stratified mortality analysis by five age groups (under 5, 5-14, 15-49, 50-64, and over 65 years). The age-group boundaries were chosen to separate groups of public health importance and different patterns of mortality. There were no major changes in age group composition over the course of the study, and age-standardized mortality rates did not differ significantly on a year-to-year basis, therefore crude mortality rates are reported throughout. Under-5 mortality rates were expressed as deaths per person-years observed, rather than the customary deaths per live births, for consistent comparison to the mortality rates in the other age groups. Exact Poisson confidence intervals were calculated for all-cause mortality rates and cause-specific mortality rates and fractions where those were based on the single-cause physician-coded causes [35]. Confidence intervals for cause-specific mortality rates and fractions in the case of the InterVA-coded causes were calculated with R [36] using bootstrapping. Cause-specific mortality rates and fractions were based on the InterVA-determined causes unless otherwise stated. The kappa analysis was done using STATA v11 [37].

Ethical approval for the Africa Centre Demographic Surveillance was provided by the University of KwaZulu-Natal Bio-Medical Research Ethics Committee.

## Results

### Mortality

**Figure 1:**
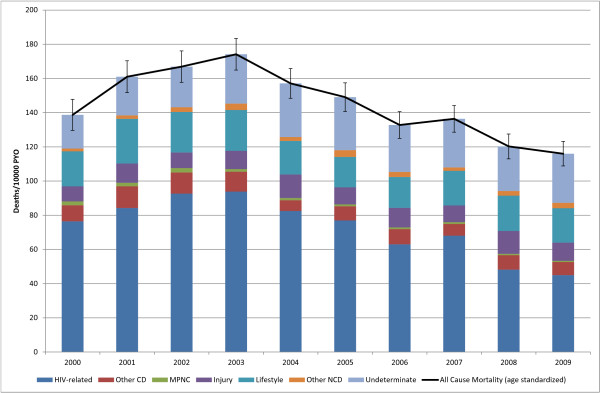
**Contribution Of Each Cause to All-Cause Mortality By Year**. (Deaths 11, 281, Person Years Observed (PYO) 784, 274).

There were 11,281 deaths over 784,274 person years of observation of 125,658 individuals between Jan. 1, 2000 and Dec. 31, 2009 (Table 2). All causes of death were coded using InterVA; the 10,267 deaths between Jan. 1, 2000 and Dec. 31, 2008, were physician-coded as well. All-cause age-standardized mortality (SMR) for the total population changed from 139 (95% CI: 130, 148) deaths per 10,000 person-years observed (PYO) in 2000 to a maximum of 174 (95% CI: 165, 183) in 2003 and then declined to 116 (95% CI: 109, 123) in 2009 (Figure 1). HIV-related causes were responsible for 50% of the deaths over the period from 2000 to 2009, followed by indeterminate causes at 18% and lifestyle-related noncommunicable diseases at 15% of all deaths. HIV-related causes were responsible for a maximum 56% of deaths in 2002, declining to a minimum of 39% of deaths in 2009. Indeterminate causes were responsible for a minimum of 14% of deaths during 2000, rising to a maximum of 25% in 2009.

**Table 2:** Person-years observed, number of deaths, and sum of InterVA likelihood values per cause of death by age group and year

0-4 yr	PYO	Deaths	HIV-related	MPNC	Injuries	Lifestyle	Other NCD	Other CD	Indeterminate
2000	9,304	185	93.7	9.2	2.4	0.3	-	40.5	38.9
2001	9,761	202	95.4	10.2	0.7	2.4	-	57.4	35.9
2002	9,969	216	87.5	13.7	5.5	-	-	64.8	44.5
2003	9,806	201	71.3	4.1	1.7	1.9	-	60.3	61.8
2004	9,837	140	38.1	4.9	1.9	-	-	30.0	65.1
2005	9,939	141	38.0	3.5	0.7	-	-	47.6	51.1
2006	10,127	118	35.5	4.2	1.9	0.6	0.8	48.6	25.5
2007	10,508	109	36.0	3.8	2.3	0.7	-	39.9	26.3
2008	10,539	118	28.6	4.7	3.0	0.7	-	53.9	27.1
2009	10,483	102	12.2	1.0	3.2	0.5	-	50.8	34.4

**5-14 yr**	**PYO**	**Deaths**	**HIV-related**	**MPNC**	**Injuries**	**Lifestyle**	**Other NCD**	**Other CD**	**Indeterminate**

2000	19,642	22	3.2	-	6.8	1.7	1.4	4.7	4.3
2001	20,723	31	13.7	-	4.5	2.6	0.1	4.6	5.5
2002	21,710	35	13.0	-	4.0	1.0	1.6	8.6	6.9
2003	22,218	41	14.2	-	10.5	2.2	0.6	2.8	10.7
2004	22,416	49	25.5	-	10.6	3.0	-	1.6	8.4
2005	22,181	38	18.1	-	6.9	2.7	-	1.3	8.9
2006	22,052	33	15.7	-	2.8	1.0	0.7	5.4	7.4
2007	22,078	27	11.8	-	4.5	0.6	-	4.7	5.5
2008	21,697	36	8.3	-	9.2	2.0	1.8	4.1	9.7
2009	21,288	22	12.9	-	3.2	0.2	-	1.6	4.1

**15-49 yr**	**PYO**	**Deaths**	**HIV-related**	**MPNC**	**Injuries**	**Lifestyle**	**Other NCD**	**Other CD**	**Indeterminate**

2000	28,618	441	327.4	4.1	34.9	32.3	0.3	5.2	36.8
2001	32,154	547	394.0	2.4	56.1	30.7	2.2	9.7	52.0
2002	35,475	625	485.4	2.6	44.2	29.5	3.2	6.6	53.5
2003	37,350	701	523.1	5.8	55.2	28.1	7.2	10.2	71.5
2004	39,059	694	480.6	3.1	81.2	26.3	5.1	4.3	93.3
2005	40,277	647	438.2	5.2	60.2	29.9	7.2	6.4	100.1
2006	41,826	580	375.6	2.1	73.2	26.2	3.3	7.4	92.3
2007	43,399	624	413.2	4.7	62.3	28.5	3.4	6.9	105.1
2008	44,235	509	294.6	1.8	85.4	23.7	6.3	4.9	92.3
2009	45,236	495	274.2	4.7	73.3	26.0	6.3	6.1	104.6

**50-64 yr**	**PYO**	**Deaths**	**HIV-related**	**MPNC**	**Injuries**	**Lifestyle**	**Other NCD**	**Other CD**	**Indeterminate**

2000	4,325	124	49.3	-	10.8	36.4	4.4	3.5	19.6
2001	4,433	153	62.6	-	14.2	49.2	4.3	3.9	18.9
2002	4,553	125	60.7	-	4.2	40.0	1.1	2.8	16.3
2003	4,666	159	70.0	-	8.1	46.5	8.4	2.8	23.3
2004	4,735	136	64.1	-	3.0	30.5	6.5	3.4	28.5
2005	4,714	167	84.8	-	5.9	35.6	8.2	2.7	29.8
2006	4,715	164	60.7	-	7.8	43.1	6.6	4.7	41.2
2007	4,836	169	74.2	-	5.0	41.6	3.3	2.9	41.9
2008	4,990	155	55.7	-	7.0	45.5	9.6	3.7	33.5
2009	5,152	174	62.1	-	6.8	46.7	10.8	4.2	43.5

**65+ yr**	**PYO**	**Deaths**	**HIV-related**	**MPNC**	**Injuries**	**Lifestyle**	**Other NCD**	**Other CD**	**Indeterminate**

2000	3,059	139	28.4	1.6	2.8	64.0	4.3	8.0	29.9
2001	3,134	196	24.8	1.9	3.6	98.3	7.7	13.4	46.3
2002	3,170	226	34.8	2.8	8.9	103.8	14.2	7.9	53.5
2003	3,187	216	31.7	1.6	5.2	102.2	12.1	12.2	51.0
2004	3,268	202	33.2	2.5	10.1	92.8	5.8	9.1	48.5
2005	3,396	198	36.0	-	6.1	74.2	15.9	8.0	57.8
2006	3,494	197	30.2	1.8	8.7	77.1	13.2	7.1	59.0
2007	3,526	229	42.6	0.7	8.8	100.6	10.1	3.8	62.4
2008	3,539	212	25.3	0.4	9.8	104.7	6.0	5.7	60.1
2009	3,474	211	28.0	1.1	5.9	100.6	10.4	3.3	61.7

**Figure 2:**
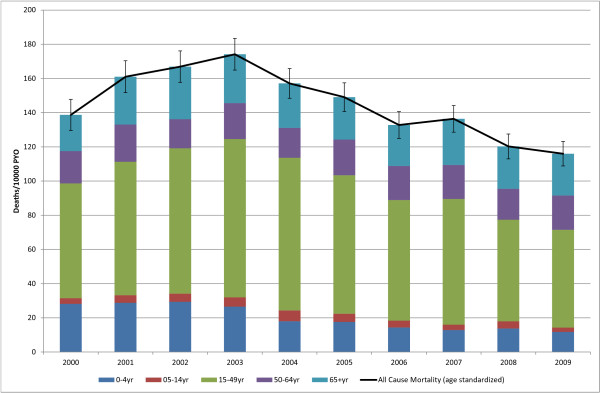
**Contribution Of Each Age Group to All-Cause Mortality By Year**. (Deaths 11, 281, Person Years Observed (PYO) 784, 274).

The largest proportion (52%) of deaths occurred in the 15-49 age group followed by the 65 and older age group at 18%. The under-5 age group contributed 20% of the deaths in 2000, but this declined to between 9% and 11% from 2007 to 2009. Overall, 82% of all deaths occurred before the age of 65 years, declining from a high of 85% in 2000 to 79% in 2009 (Figure 2).

**Figure 3:**
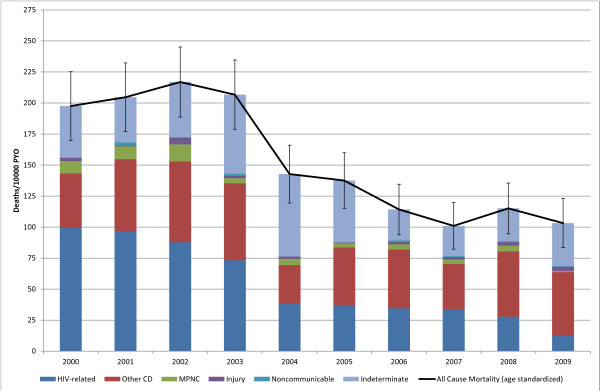
**< 5 yr Age Standardized Mortality**. (Deaths 1, 531, Person Years Observed (PYO) 100, 274).

**Figure 4:**
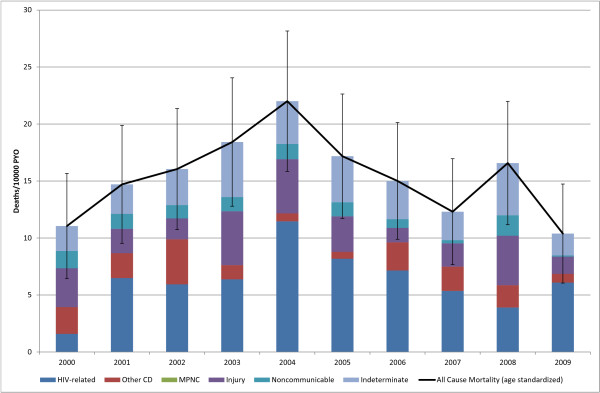
**5-14 yr Age Standardized Mortality**. (Deaths 333, Person Years Observed (PYO) 216, 005).

The under-5 all-cause SMR peaked at 217 (95% CI: 189, 245) deaths per 10,000 PYO in 2002 and then declined rapidly to 103 (95% CI: 83,103) in 2009 (Figure 3). This is greater than a twofold decline in mortality. HIV-related mortality (Table 3) in this age group had a high in 2000 of 101 deaths per 10,000 PYO (95% CI: 86,115), plateaued at around 37 deaths per 10,000 PYO between 2004 and 2007, and then declined further to a low of 12 (95% CI: 6, 17) in 2009. Other communicable disease SMR varied between 31 (95%: CI 22, 38) in 2004 and 65 (95% CI: 54, 76) deaths per 10,000 PYO in 2002. Mortality due to indeterminate causes was high in this age group varying around 41 deaths per 10,000 PYO and peaked around 60 deaths per 10,000 between 2003 and 2005. The cause-specific mortality fraction (CSMF) due to HIV-related causes declined from 51% (95% CI: 44, 58) in 2000 to 12% (95% CI: 6, 17) in 2009. As a result of the overall decline in the mortality rate in this age group, the CSMF for other communicable diseases increased from 22% (95% CI: 17, 27) in 2000 to 50% (95% CI: 42, 57) in 2009. The all-cause SMR for the 5-14 age group peaked at 22 (95% CI: 16, 28) deaths per 10,000 PYO in 2004 and then declined to 10 (95% CI: 6, 15) in 2009 (Figure 4).

**Table 3:** Cause-specific mortality rates by age group and year (per 10,000 person years observed)

2000	0-4 yrs (95% CI)	5-14 yrs (95% CI)	15-49 yrs (95% CI)	50-64 yrs (95% CI)	65+ (95% CI)
HIV-related	100.8 (88.1-113.9)	1.6 (0.0-3.1)	114.3 (109.1-119.5)	113.8 (89.5-137.0)	94.3 (65.6-122.2)
MPNC	8.0 (2.8-12.9)	-	1.4 (0.1-2.5)	-	3.9 (0.0-7.6)
Injuries	2.6 (0.0-5.2)	3.5 (1.4-5.3)	11.9 (8.2-15.6)	24.4 (9.6-36.7)	9.3 (0.0-16.8)
Lifestyle	0.3 (0.0-0.7)	0.9 (0.0-1.8)	11.1 (7.6-14.4)	83.8 (62.8-103.7)	211.2 (180.8-240.9)
Other NCD	-	0.7 (0.0-1.4)	0.2 (0.0-0.5)	11.1 (2.6-18.0)	18.1 (3.8-29.5)
Other CD	45.0 (35.4-54.5)	2.4 (0.3-4.2)	1.6 (0.3-2.7)	5.5 (0.0-11.5)	22.0 (11.0-32.8)
Indeterminate	42.1 (33.7-49.1)	2.2 (1.2-3.0)	13.4 (11.1-15.6)	48.2 (35.9-59.0)	95.6 (78.0-113.6)

**2001**	**0-4 yrs (95% CI)**	**5-14 yrs (95% CI)**	**15-49 yrs (95% CI)**	**50-64 yrs (95% CI)**	**65+ (95% CI)**

HIV-related	97.7 (84.6-110.8)	6.6 (4.4-9.1)	122.9 (116.9-128.9)	143.9 (118.4-170.3)	77.3 (50.9-102.3)
MPNC	10.0 (4.1-15.2)	-	0.4 (0.0-0.9)	-	6.1 (0.0-12.2)
Injuries	0.7 (0.0-1.4)	2.2 (0.4-3.7)	17.2 (12.5-21.5)	31.9 (15.7-47.4)	10.3 (0.0-19.3)
Lifestyle	2.5 (0.1-4.5)	1.5 (0.0-2.7)	9.9 (6.5-12.6)	111.4 (87.4-135.0)	324.3 (283.6-365.0)
Other NCD	-	0.1 (0.0-0.1)	0.8 (0.0-1.5)	9.7 (1.1-16.6)	24.4 (8.5-38.4)
Other CD	59.2 (46.6-70.2)	2.2 (0.4-3.6)	2.9 (1.0-4.6)	7.2 (0.0-12.9)	38.2 (21.3-54.6)
Indeterminate	36.9 (29.3-43.0)	2.5 (1.3-3.4)	15.9 (13.4-18.2)	41.1 (28.8-52.3)	144.8 (121.5-168.7)

**2002**	**0-4 yrs (95% CI)**	**5-14 yrs (95% CI)**	**15-49 yrs (95% CI)**	**50-64 yrs (95% CI)**	**65+ (95% CI)**

HIV-related	87.7 (74.6-101.9)	5.9 (3.6-8.3)	137.1 (132.1-142.0)	134.7 (111.5-156.7)	106.8 (73.3-138.1)
MPNC	12.8 (6.3-18.0)	-	0.7 (0.0-1.5)	-	8.8 (0.9-15.1)
Injuries	5.5 (1.3-9.2)	1.8 (0.0-3.4)	12.0 (8.5-15.3)	7.8 (0.0-14.3)	26.3 (9.0-41.1)
Lifestyle	-	0.5 (0.0-1.0)	8.3 (5.2-10.9)	88.0 (66.2-108.8)	341.3 (298.3-388.4)
Other NCD	-	0.7 (0.0-1.5)	0.9 (0.0-1.7)	2.4 (0.0-4.9)	46.9 (27.1-65.0)
Other CD	65.6 (54.5-76.9)	4.0 (1.9-5.8)	1.8 (0.4-3.0)	5.5 (0.0-10.6)	22.7 (7.9-35.8)
Indeterminate	45.0 (37.4-51.7)	3.2 (1.7-4.4)	15.3 (13.0-17.6)	36.2 (23.9-46.9)	160.3 (132.6-187.5)

**2003**	**0-4 yrs (95% CI)**	**5-14 yrs (95% CI)**	**15-4 9 yrs (95% CI)**	**50-64 yrs (95% CI)**	**65+ (95% CI)**

HIV-related	72.7 (58.6-85.8)	6.4 (3.8-8.9)	140.6 (135.7-145.5)	148.2 (123.6-172.4)	99.3 (67.9-126.6)
MPNC	4.1 (0.2-7.4)	-	1.4 (0.0-2.4)	-	5.0 (0.0-9.6)
Injuries	1.8 (0.0-3.5)	4.8 (2.5-7.0)	13.8 (10.4-17.1)	17.4 (3.6-27.5)	16.3 (0.8-28.0)
Lifestyle	1.9 (0.0-3.6)	0.9 (0.0-1.8)	8.3 (5.8-10.7)	105.5 (81.3-127.6)	330.0 (292.1-369.7)
Other NCD	-	0.3 (0.0-0.6)	1.9 (0.6-3.1)	18.4 (7.4-28.2)	39.6 (18.2-58.8)
Other CD	61.6 (49.5-73.0)	1.3 (0.1-2.3)	2.7 (1.2-4.1)	5.0 (0.0-9.6)	31.3 (16.0-44.9)
Indeterminate	62.9 (51.6-73.0)	4.9 (2.9-6.6)	18.9 (16.6-21.1)	46.3 (36.7-55.4)	156.2 (131.2-181.2)

**2004**	**0-4 yrs (95% CI)**	**5-14 yrs (95% CI)**	**15-49 yrs (95% CI)**	**50-64 yrs (95% CI)**	**65+ (95% CI)**

HIV-related	38.7 (27.8-49.1)	11.4 (8.7-14.3)	123.8 (118.5-129.4)	130.7 (111.0-151.6)	100.9 (71.1-129.3)
MPNC	4.6 (0.3-7.9)	-	0.8 (0.0-1.5)	-	6.3 (0.0-11.2)
Injuries	1.9 (0.0-3.8)	4.8 (2.3-6.9)	20.1 (15.9-23.8)	6.3 (0.0-12.5)	24.2 (8.9-39.0)
Lifestyle	-	1.3 (0.0-2.6)	6.8 (4.7-9.0)	71.8 (53.4-88.5)	302.2 (266.5-337.7)
Other NCD	-	-	1.3 (0.3-2.2)	13.8 (4.3-21.7)	18.4 (4.7-30.4)
Other CD	30.5 (23.0-38.9)	0.7 (0.0-1.4)	1.0 (0.2-1.7)	5.2 (0.0-9.4)	19.8 (8.0-29.9)
Indeterminate	66.6 (55.9-76.7)	3.6 (1.8-5.2)	23.9 (20.5-27.2)	59.6 (45.6-72.5)	146.1 (122.4-169.3)

**2005**	**0-4 yrs (95% CI)**	**5-14 yrs (95% CI)**	**15-49 yrs (95% CI)**	**50-64 yrs (95% CI)**	**65+ (95% CI)**

HIV-related	38.3 (29.5-46.9)	8.2 (5.3-10.9)	108.9 (103.7-114.6)	179.4 (154.0-201.9)	104.3 (74.4-131.6)
MPNC	3.6 (0.2-6.3)	-	1.2 (0.3-2.0)	-	-
Injuries	0.7 (0.0-1.4)	3.1 (1.1-5.0)	14.5 (11.0-17.5)	12.5 (2.0-21.0)	17.8 (3.9-30.0)
Lifestyle	-	1.2 (0.0-2.4)	7.9 (5.3-10.2)	78.7 (59.2-97.4)	232.4 (199.1-264.9)
Other NCD	-	-	1.8 (0.8-2.7)	17.6 (5.7-27.3)	52.3 (30.9-73.2)
Other CD	47.9 (38.7-57.3)	0.6 (0.0-1.2)	1.5 (0.5-2.4)	4.0 (0.0-7.4)	17.8 (4.6-27.8)
Indeterminate	51.4 (43.0-58.6)	4.0 (2.1-5.9)	24.8 (21.2-27.9)	62.1 (46.8-76.9)	158.3 (130.4-183.0)

**2006**	**0-4 yrs (95% CI)**	**5-14 yrs (95% CI)**	**15-49 yrs (95% CI)**	**50-64 yrs (95% CI)**	**65+ (95% CI)**

HIV-related	35.1 (26.2-42.8)	7.1 (5.0-9.5)	89.8 (84.8-95.0)	126.8 (104.5-149.4)	86.8 (57.1-111.7)
MPNC	4.1 (0.0-7.6)	-	0.5 (0.0-0.9)	-	4.7 (0.0-8.7)
Injuries	1.8 (0.0-3.7)	1.3 (0.0-2.5)	16.8 (12.7-20.5)	14.9 (2.0-24.4)	24.9 (7.5-38.5)
Lifestyle	0.6 (0.0-1.2)	0.4 (0.0-1.0)	6.2 (4.2-8.1)	95.0 (74.9-114.4)	224.2 (190.7-257.3)
Other NCD	0.8 (0.0-1.6)	0.3 (0.0-0.7)	0.8 (0.0-1.4)	13.9 (4.2-21.4)	39.4 (21.0-57.1)
Other CD	47.9 (38.9-56.6)	2.5 (0.7-3.8)	1.5 (0.5-2.3)	9.5 (2.4-15.2)	18.1 (8.1-27.6)
Indeterminate	26.2 (20.5-31.8)	3.8 (1.3-5.7)	23.0 (19.9-26.2)	87.7 (73.2-102.6)	165.8 (140.4-189.7)

**2007**	**0-4 yrs (95% CI)**	**5-14 yrs (95% CI)**	**15-49 yrs (95% CI)**	**50-64 yrs (95% CI)**	**65+ (95% CI)**

HIV-related	34.3 (25.1-42.8)	5.4 (3.3-7.5)	95.4 (91.1-100.3)	153.4 (130.8-177.3)	116.3 (85.5-144.9)
MPNC	3.0 (0.0-5.5)	-	1.4 (0.2-2.3)	-	2.0 (0.0-4.3)
Injuries	2.2 (0.0-4.1)	2.0 (0.4-3.5)	13.7 (10.4-16.8)	10.4 (1.1-18.1)	24.1 (10.0-36.3)
Lifestyle	0.7 (0.0-1.5)	0.3 (0.0-0.6)	6.4 (4.3-8.4)	88.9 (66.7-109.4)	296.1 (261.9-328.5)
Other NCD	-	-	0.8 (0.1-1.4)	5.6 (0.0-10.1)	29.5 (14.4-42.7)
Other CD	38.6 (29.9-47.2)	2.1 (0.2-3.7)	1.6 (0.6-2.5)	4.3 (0.0-8.2)	8.3 (1.3-14.3)
Indeterminate	25.1 (19.9-30.4)	2.5 (0.8-3.9)	24.5 (20.9-27.5)	86.9 (68.2-103.5)	173.3 (144.5-199.4)

**2008**	**0-4 yrs (95% CI)**	**5-14 yrs (95% CI)**	**15-49 yrs (95% CI)**	**50-64 yrs (95% CI)**	**65+ (95% CI)**

HIV-related	27.1 (18.5-35.7)	3.8 (1.6-6.0)	66.9 (62.4-72.1)	111.7 (89.1-134.3)	70.5 (46.9-93.4)
MPNC	4.4 (0.5-7.6)	-	1.0 (0.1-1.8)	-	0.3 (0.0-0.7)
Injuries	2.8 (0.0-5.1)	4.2 (1.9-6.4)	18.7 (14.7-22.4)	14.7 (2.1-24.6)	27.7 (10.8-41.2)
Lifestyle	0.7 (0.0-1.5)	0.9 (0.0-1.9)	5.3 (3.4-7.1)	96.7 (77.8-116.4)	295.6 (260.9-333.1)
Other NCD	-	1.1 (0.0-2.1)	1.6 (0.6-2.5)	19.8 (6.3-30.2)	19.7 (6.8-31.1)
Other CD	51.2 (42.8-60.0)	1.7 (0.2-3.0)	1.0 (0.1-1.8)	7.5 (0.0-13.6)	14.5 (4.9-23.1)
Indeterminate	25.8 (19.4-31.5)	4.8 (2.8-6.5)	20.5 (17.5-23.3)	60.3 (44.7-74.0)	173.6 (145.9-197.4)

**2009**	**0-4 yrs (95% CI)**	**5-14 yrs (95% CI)**	**15-49 yrs (95% CI)**	**50-64 yrs (95% CI)**	**65+ (95% CI)**

HIV-related	11.7 (6.2-16.7)	5.6 (3.6-7.7)	58.3 (53.9-62.3)	114.9 (94.5-137.2)	78.3 (51.7-103.4)
MPNC	0.9 (0.0-2.1)	-	1.2 (0.2-2.1)	-	3.1 (0.0-6.2)
Injuries	3.0 (0.0-5.7)	1.5 (0.0-2.9)	15.4 (11.7-18.9)	12.3 (1.6-20.9)	15.7 (2.6-27.0)
Lifestyle	0.4 (0.0-0.9)	0.1 (0.0-0.2)	5.3 (3.3-7.0)	94.5 (74.9-113.8)	285.8 (249.9-322.0)
Other NCD	-	-	1.5 (0.4-2.3)	20.6 (8.4-30.6)	29.2 (14.4-42.9)
Other CD	45.2 (38.2-52.2)	0.8 (0.0-1.5)	1.1 (0.2-1.8)	6.2 (0.3-11.0)	9.6 (0.9-16.9)
Indeterminate	36.1 (29.0-44.0)	2.3 (1.0-3.5)	26.9 (23.7-30.1)	89.4 (70.0-106.1)	185.6 (157.9-212.9)

**Figure 5:**
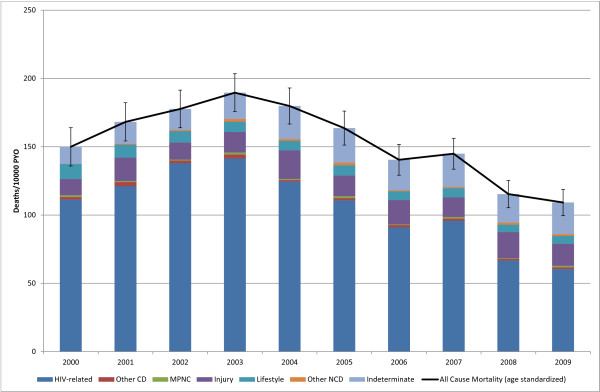
**15-49 yr Age Standardized Mortality**. (Deaths 5, 863, Person Years Observed (PYO) 387, 630).

The all-cause SMR for the 15-49 age group peaked at 190 (95% CI: 176, 203) deaths per 10,000 PYO in 2003 and then declined to 109 (95% CI: 100, 119) in 2009 (Figure 5). HIV-related mortality peaked in 2003 at 140 (95% CI: 135, 145) deaths per 10,000 PYO and then declined to 61 (95% CI: 56, 65) in 2009 (Table 3). The CSMF for HIV-related causes declined from 74% (95% CI: 71, 78) in 2000 to 55% (95% CI: 52, 59) in 2009. This decline in HIV-related CSMF appears to be at the expense of an increase in indeterminate CSMF, which increased over the same period from 8% (95% CI: 7, 10) to 21% (95% CI: 19, 24).

**Figure 6:**
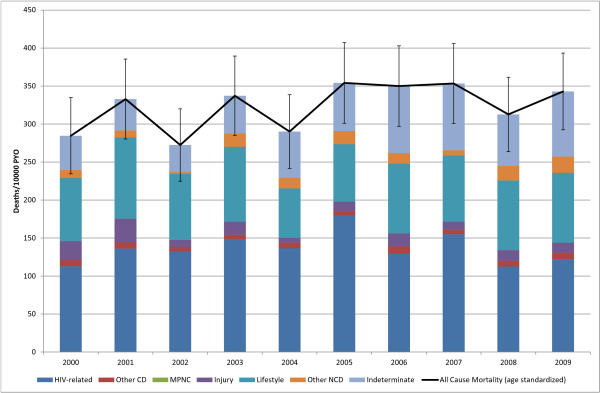
**50-64 yr Age Standardized Mortality**. (Deaths 1, 526, Person Years Observed (PYO) 47, 118).

There was no discernible trend in all-cause mortality in the 50-64 age group (Figure 6). HIV-related causes constituted the largest CSMF at 42% for the entire period, followed by lifestyle-related noncommunicable diseases at 27% and indeterminate causes at 19%. There was no significant trend in HIV-related mortality and the highest mortality rates observed occurred in 2005 (180 (95% CI: 156, 206)) and 2007 (154 (95% CI: 129, 179)) after the introduction of an antiretroviral therapy (ART) program in the area in 2004 (Table 3). Lifestyle-related noncommunicable mortality remained stable at around 88 deaths per 10,000 PYO.

**Figure 7:**
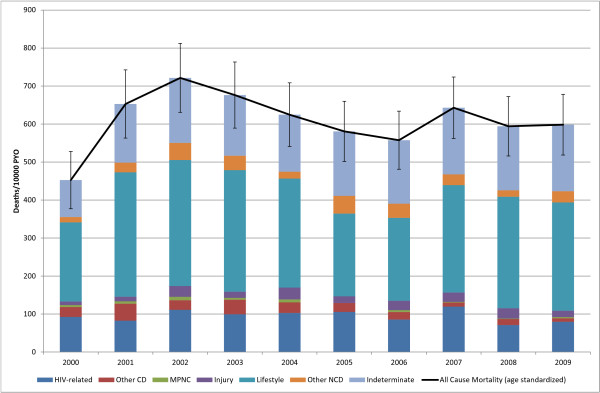
**65+yr Age Standardized Mortality**. (Deaths 1, 526, Person Years Observed (PYO) 47, 118).

Age-standardized mortality in the over-65 age group appeared to increase in the early part of the decade, rising from 453 (95% CI: 377, 528) deaths per 10,000 in 2000 to a peak of 721 (95% CI: 631,812) in 2002 and plateauing off to remain stable at around 611 deaths per 10,000 PYO (Figure 7). Noncommunicable diseases were responsible for the largest CSMF at 50%, with lifestyle-related CSMF at 45%. Indeterminate (26%) and HIV-related (16%) causes were also important.

### Comparison of physician with InterVA coding

There was substantial agreement (79% kappa = 0.68 (95% CI: 0.67, 0.69)) between physician coding and InterVA coding at the burden of disease group level. This agreement varied significantly among age groups (Table 4), with the lowest agreement in the under-5 age group (kappa = 0.44 (95% CI: 0.41, 0.47)) and the over-65 age group (kappa = 0.50 (95% CI: 0.48, 0.52)), with the remaining age groups around the overall agreement level. There were no significant changes in agreement over time (Table 5).

**Table 4:** Agreement between physician and InterVA cause of death allocation by age group

Age Group	Agreement	Kappa (95% confidence interval)	p
0-4	61%	0.43 (0.40-0.46)	< 0.0001
5-14	76%	0.68 (0.63-0.74)	< 0.0001
15-49	86%	0.71 (0.70-0.72)	< 0.0001
50-64	77%	0.68 (0.65-0.70)	< 0.0001
65+	68%	0.50 (0.48-0.53)	< 0.0001

Overall	79%	0.68 (0.67-0.69)	< 0.0001

**Table 5:** Agreement between physician and InterVA cause of death allocation by year

Year	Agreement	Kappa (95% confidence interval)	p
2000	78%	0.67 (0.64-0.70)	< 0.0001
2001	82%	0.72 (0.69-0.74)	< 0.0001
2002	82%	0.71 (0.68-0.74)	< 0.0001
2003	78%	0.66 (0.63-0.69)	< 0.0001
2004	77%	0.66 (0.63-0.69)	< 0.0001
2005	75%	0.63 (0.61-0.66)	< 0.0001
2006	76%	0.66 (0.63-0.69)	< 0.0001
2007	79%	0.69 (0.66-0.72)	< 0.0001
2008	79%	0.72 (0.69-0.75)	< 0.0001

## Discussion

Cause-specific mortality trends in the under-5 age group and the 15-49 age group were dominated by trends in HIV-related mortality in response to the introduction of interventions such as preventing mother-to-child transmission of HIV (PMTCT) and ART [7,8]. There were no significant trends in other causes of mortality in these age groups. The CSMF for HIV-related causes for the under-5 age group (34%) for the 2002-2005 period was higher than the value (26%) reported by Byass [20] using InterVA (with the same set of input indicators as used by this study) for the same period in Agincourt, a demographic surveillance site in the Mpumalanga province of South Africa. In the case of the 15-49 age group, the HIV-related CSMF (72%) in this study was also higher than the value (54%) reported for the Agincourt site.

In the older age groups, HIV-related mortality remained important but showed no significant decline in the second half of the 2000-2009 period. Noncommunicable-disease mortality increased with increasing age and was the major cause of death in the 65-and-older age group. There were no clear trends in noncommunicable disease mortality over time.

The proportions of indeterminate causes in this study for the different age groups were generally lower than the levels reported by Byass [20] for the Agincourt site, with the exception of the under-5 age group. In the under-5 age group, 32% in this study compared to 26% in the Byass study; 21% in this study compared to 38% in the Byass study in the 5-14 age group; 12% in this study compared to 26% in the Byass study in the 15-49 age group; 17% in this study compared to 31% in the Byass study in the 50-64 age group, and 25% in this study compared to 33% in the Byass study in the 65-and-older age group.

The verbal autopsy questionnaire was not designed from inception with the InterVA input indicators in mind. A number (18 out of 106 indicators) did not map directly to the questionnaire and had to be derived indirectly. Data quality in relation to the InterVA input indicators could not be monitored on an ongoing basis and some of the changes over time in the indeterminate-cause proportion could reflect data-quality issues.

## Conclusions

There has been a substantial decline in proportion of deaths due to HIV-related causes in the under-5 age group over the study period coupled with a fairly constant mortality rate due to other communicable diseases and indeterminate causes. This would indicate that further gains in reducing under-5 mortality would require investigation of causes other than HIV and possibly changes in public health services. Further research is required to determine whether the resilience to change in other communicable disease mortality is due to interaction between interventions aimed at HIV and those aimed at other causes of child mortality, or due to unrelated factors.

In the 15-49 age group, the positive impact of HIV-related interventions was substantial. We did not explore sex-specific mortality, but given the different age pattern in HIV prevalence [27], one would expect some differences in the sex-specific HIV-related mortality trends. Although trauma mortality is dwarfed by HIV-related mortality in this age group, it is still considerably higher than the global mortality estimate [38].

In the older age groups, the dual burdens of communicable and noncommunicable diseases were evident. The lack of a substantial decline in HIV-related mortality in those over 50 years old requires further investigation to determine whether this was due to lack of access or response to treatment programs or an artifact of competing risks from other mortality causes.

The InterVA verbal autopsy program performed well, and the conclusions based on InterVA mortality cause allocation would have been no different had they been based on physician mortality cause allocation. The InterVA program allowed more timely analysis of cause-specific mortality; as a result we could include the 2009 deaths in this analysis in spite of the fact that physician coding is not yet complete for 2009.

Verbal autopsy based methods enabled the timely measurement of changing trends in cause-specific mortality to provide policymakers with the much-needed information to allocate resources to appropriate health interventions.

## Competing interests

The authors declare that they have no competing interests.

## Authors' contributions

AJH was responsible for the data analysis and drafting the manuscript. TM supervised the verbal autopsy data collection and participated in the data analysis. MLN is the director of the Africa Centre and contributed to the drafting and reviewing of the manuscript. All authors have read and approved the final manuscript.
